# The network structure of psychological wellbeing: paranormal belief is peripheral but meaningful

**DOI:** 10.3389/fpsyg.2026.1846072

**Published:** 2026-06-04

**Authors:** Neil Dagnall, Andrew Denovan, Robert C. Dempsey, Kenneth Graham Drinkwater, Stephen Walsh, Alex Escolá Gascón

**Affiliations:** 1 School of Psychology, Manchester Metropolitan University, Manchester, United Kingdom; 2 School of Psychology, Liverpool John Moores University, Liverpool, United Kingdom; 3 Department of Quantitative Methods and Statistics, Universidad Pontificia Comillas, Madrid, Spain

**Keywords:** flourishing, network analysis, optimism, paranormal belief, psychological wellbeing, thriving

## Abstract

**Introduction:**

Paranormal belief (PB) remains prevalent within modern societies despite the dominance of scientific rationalism. While early research conceptualized PB as maladaptive, contemporary perspectives suggest it serves adaptive, meaning-making functions. However, the structural role of PB within broader systems of psychological wellbeing remains unclear. Addressing this gap, the present study employed network analysis to examine relationships between PB, affect (positive and negative), optimism, pessimism, and eudaimonic wellbeing (thriving and flourishing).

**Methods:**

A sample of 1,430 UK adults completed validated self-report measures. A Gaussian Graphical Model with EBICglasso regularization was estimated to identify conditional associations and centrality indices within the network. Centrality indices (strength, closeness, betweenness, and expected influence) determined the relative importance and bridging roles of nodes.

**Results:**

The network revealed a highly interconnected wellbeing structure centered on thriving, flourishing, and positive affect. Optimism emerged as a key bridge node linking positive and negative domains. PB occupied a peripheral position, demonstrating weak but positive associations with optimism, negative affect, and, to a lesser extent, thriving, while showing no direct connections with flourishing or positive affect.

**Discussion:**

Findings indicate that PB is not centrally embedded within the wellbeing system but operates as a peripheral interpretive framework indirectly linked to adaptive functioning. Specifically, PB contributes to psychological wellbeing through its association with optimism and future-oriented expectations. Overall, the results support contemporary models that position PB as a non-pathological, context-dependent cognitive framework. Implications for understanding the role of belief systems in meaning-making and psychological adaptation are discussed.

## Introduction

Despite the prominence of scientific rationalism and increased secularization, paranormal belief (PB) prevails within modern societies. This is evidenced by academic surveys (Dagnall et al., 2016) and opinion polls (IPSOS MORI, 1998, 2003; Newport and Strausberg, 2001; Moore, 2005), which report high endorsement levels. While comparing results is difficult due to sampling constraints and use of different measurement instruments, theorists approximate incidence of PB in Western non-clinical populations at approximately 50% (Irwin, 2009; Marks, 2021). Such estimates demonstrate that PB is a common feature of human cognition (Bering, 2006). Moreover, they suggest that for some individuals that PB fulfills beneficial functions (Kennedy et al., 1994; Irwin, 2009).

This perspective represents a theoretical advance from foundational scholarly work linking PB with poor psychological functioning. This view was informed by negative perceptions of scientifically unsubstantiated notions and supported by rudimentary empirical findings that identified positive correlations between PB and psychopathological outcomes (e.g., psychiatric distress, Peltzer, 2002); and depressive and manic symptoms (Thalbourne and French, 1995; Thalbourne and Storm, 2019). In this context, researchers interpreted PB as the byproduct of deficient or impaired cognitive processes. A stance embodied within the deficit model (Alcock, 1981), which considers PB as maladaptive and affiliated with reduced subjective wellbeing.

Noting the prosaic nature of PB and the limitations of prior research, particularly the focus on specific facets (i.e., superstition, spiritualism, and precognition), recent studies have reappraised the nature of PB. This research has reconceptualized PB as a heterogeneous construct, whereby the psychological impact of PB (i.e., adaptive value) is influenced by interactions with cognitive and affective factors.

This nuanced relationship is demonstrated by person-centered analytical approaches that identify distinct psychological profiles within samples. Specifically, evidence from latent profile analysis (LPA) found that reduced wellbeing (i.e., heightened stress, somatic complaints, and lower life satisfaction) was associated with profiles where PB co-occurred with psychopathology-related traits (i.e., schizotypy, depression, and manic-depressive tendencies) (Dagnall et al., 2022a; Drinkwater et al., 2024). Within these profiles, transliminality and cognitive disorganization acted as bridging mechanisms increasing vulnerability to negative outcomes (Dagnall et al., 2022a,c). Additionally, PB was associated with low self-esteem and poorer psychological health (Dagnall et al., 2025c).

Thus, in the absence of psychopathological factors, PB functions as a benign or adaptive aspect of cognition (Goulding, 2005). This is especially true in non-clinical populations since PB does not exert a negative effect on wellbeing when levels of psychopathology are low (Drinkwater et al., 2024). Moreover, PB and schizotypy show discrete relationships with psychological health. While schizotypy is linked to diminished positive wellbeing (Claridge, 1997), PB is positively associated with greater life satisfaction and sense of meaning (Dagnall et al., 2024). This distinction suggests that, for a substantial proportion of people, PB operates as a stable and non-detrimental cognitive framework (Goulding, 2005; Irwin, 2009).

This operationalization is supported by longitudinal and network analytic research, which reports that PB, by correlating positively with presence of and the search for meaning in life, enhances wellbeing (Dagnall et al., 2025b). When these dimensions work in combination, search reinforces or strengthens presence (Steger et al., 2006). This interaction is important because the constituent components of meaning in life (i.e., purpose, coherence, and personal significance) affiliate positively with wellbeing (Martela and Steger, 2016).

In this context, PB functions as a subjective interpretive framework that helps individuals navigate existential challenges, a process mediated by positive outlook (i.e., optimism) and active coping strategies (Dagnall et al., 2025b). Thus, even when linked to avoidant coping, PB enables individuals to maintain psychological equilibrium (Dagnall et al., 2025b,c). Beyond this stabilizing function, PB bolsters internal self-concept resources. Specifically, higher levels of belief are associated with increased creative self-efficacy and a stronger identity (Dagnall et al., 2025a). This, in turn, is affiliated with enhanced self-esteem, which mediates the relationship between PB and meaning in life by reinforcing the presence of meaning and individuals’ confidence in their ability to address life challenges (Dagnall et al., 2025a).

## Study rationale

To further investigate relationships between PB, wellbeing, and psychological functioning, the present study employed a multi-measurement framework. Specifically, the study examined the trajectory from hedonic affect (immediate emotional states) to eudaimonic growth (long-term flourishing and purpose). While positive and negative experiences capture everyday emotions, the inclusion of dispositional optimism and pessimism afforded insights into future-oriented expectations. This approach was grounded in the notion that PBs act as subjective, generic theories of the world (Kanazawa, 2015) that facilitate goal setting and future behavior (Betsch et al., 2021).

Despite the theoretical shift toward the instrumental nature of PB, empirical research into its salutary benefits remains scarce. Furthermore, the limited literature has relied on variable-centered approaches, which analyze PB in isolation and fail to account for its position within broader psychological systems. Consequently, it remains unclear how PB is structurally situated within networks of wellbeing or the extent to which it bridges hedonic and eudaimonic processes. To address these gaps, the present study employed network analysis (NA) to model PB alongside key affective and wellbeing variables (Dagnall et al., 2022a). This technique enables identification of central and bridging nodes and clarifies whether PB operates as an integrated or peripheral component with a eudaimonic system.

Accordingly, the inclusion of thriving and flourishing scales extended consideration of wellbeing from feeling good to functioning well and fulfillment of core psychological needs. This approach recognizes that PBs are often embedded in, and facilitate, creation of meaning (Bruner, 1993; Harari, 2015) and serve as a framework for addressing life challenges. This fact is often overlooked in Western research because supernatural notions conflict with scientific-rationalism orientations.

Building upon the NA approach utilized in previous research (e.g., Dagnall et al., 2022a), the present study mapped variable interconnectedness. By conceptualizing constructs as a dynamic network, NA provides a granular representation of how specific variables function as central or bridge nodes. This structural insight is essential for identifying the precise pathways through which paranormal-related emotional states integrate into broader adaptive outcomes. Consequently, NA offers a holistic representation of psychological health, clarifying the extent to which PBs are functionally embedded within processes of eudaimonic growth (Betsch et al., 2021).

## Methods

### Participants

The sample, recruited via Bilendi, an established provider of high-quality data (Hajek et al., 2024), comprised 1,430 UK-based adults (*M*_*age*_ = 52.27, SD = 14.01; range 18–79 years). The gender distribution consisted of 684 males (47.8%; *M*_*age*_ = 54.20, SD = 13.24), 739 females (51.7%; *M*_*age*_ = 50.65, SD = 14.40), and participants identifying as non-binary (*n* = 4), transgender (*n* = 2), or not specified (*n* = 1). The breakdown of educational attainment was 257 participants (18.0%) pre-degree qualifications, 383 (26.8%) undergraduate degrees, 279 (19.5%) postgraduate degrees, 330 (23.1%) vocational qualifications, and 181 (12.7%) professional qualifications. Overall, the sample represented a broad adult demographic.

### Materials

This study employed psychometrically validated self-report measures.

### Paranormal belief

The Revised Paranormal Belief Scale (RPBS) (Tobacyk, 2004) assessed endorsement of supernatural powers and entities. The RPBS encompasses 26 items, which appear as statements (e.g., “Black cats can bring good luck”). Participants record their responses via a seven-point Likert-type scale (1 = strongly disagree to 7 = strongly agree).

The instrument comprises seven factors focusing on Traditional Religious Belief (i.e., culturally sanctioned religious concepts; e.g., “There is a devil”), Psi (i.e., psychic abilities and mental powers; e.g., “A person’s thoughts can influence the movement of a physical object”), Witchcraft (i.e., magic and sorcery; e.g., “Black magic really exists”), Superstition (i.e., influence of specific actions or objects on outcomes; e.g., “The number “13” is unlucky”), Spiritualism (i.e., survival of consciousness and communication with the deceased; e.g., “It is possible to communicate with the dead”), Extraordinary Life Forms (i.e., scientifically unverified creatures; e.g., “The Loch Ness monster of Scotland exists”), and Precognition (i.e., knowledge of future events; e.g., “Astrology is a way to accurately predict the future”).

In the present study, the RPBS was operationalized using total (rather than subscale) scores. Theoretical and psychometric considerations informed this decision. While Tobacyk (2004) validated the RPBS as a multidimensional instrument, subsequent psychometric evaluation concludes that its lower-order structure lacks stability across samples (Lange et al., 2000). Hence, scores are best represented as a dominant general factor. This interpretation concurs with bifactor modeling, which reports that a substantial proportion of shared variance is attributable to a single overarching paranormal dimension, with comparatively limited reliable variance remaining at the subdomain level (Drinkwater et al., 2017).

The unidimensional approach also enhances statistical efficiency (i.e., avoids collinearity and redundant nodes) and construct reliability for network analysis, where a unified latent representation is preferable when the primary interest concerns global connectivity among psychological constructs rather than domain-specific belief differentiation (Borsboom and Cramer, 2013). To facilitate comparisons with related studies using Rasch-scaled totals, item scores were converted from 1–7 to 0–6. Thus, scale scores ranged from 0 to 156 with higher scores indicating greater PB.

### Positive and negative experiences

The Scale of Positive and Negative Experience (SPANE) (Diener et al., 2010) measured the frequency of positive (six items; SPANE-P; e.g., good and happy) and negative emotional experiences (six items; SPANE-N; e.g., sad and angry) over the past 4 weeks. Participants record their experiences using a five-point Likert scale ranging from 1 (very rarely or never) to 5 (very often or always). Higher scores on SPANE-P and SPANE-N indicate higher levels of positive and negative affect, respectively.

### Optimism and pessimism

Optimism–Pessimism Short Scale–2 (SOP2; Kemper et al., 2013; Nießen et al., 2022) evaluated generalized confidence and positive expectations about the future. The scale comprises two items measuring optimism and pessimism, which are accompanied by a brief conceptual definition of each construct. Participants respond via a seven-point Likert scale (1 = not at all to 7 = very), with higher scores correspondingly indicating greater optimism and pessimism.

### Thriving

The Brief Inventory of Thriving (BIT; Su et al., 2014) measured positive functioning and holistic psychological wellbeing. The instrument consists of 10 items that encompass core facets of thriving (e.g., purpose, meaning, optimism self-efficacy, and social belonging). Inventory items appear as statements (e.g., “My life is going well”) and participants indicate their level of agreement using a five-point Likert-type scale ranging from 1 (strongly disagree) to 5 (strongly agree). Item summation produces an index total, with higher totals reflecting greater psychological thriving and life satisfaction.

### Flourishing

The Flourishing Scale (FS; Diener et al., 2009) measures self-perceived success in core areas of functioning (i.e., relationships, self-esteem, purpose, and optimism). By integrating these elements, the instrument provides a comprehensive profile of psychological resources and social integration. The scale comprises eight items, presented as statements, and participants record their responses on a seven-point Likert scale. Higher scores reflect greater functioning and social-psychological wellbeing.

Each instrument has demonstrated robust reliability and validity. RPBS (Dagnall et al., 2023; Drinkwater et al., 2017), SPANE (Diener et al., 2010), SOP2 (Kemper et al., 2013; Nießen et al., 2022), BIT (Su et al., 2014), and FS (Diener et al., 2009, 2010).

### Procedure

Prospective participants received a hyperlink accompanied by a short study description. Activation of the hyperlink enabled participants to access the information hosted on Qualtrics. This detailed the study aims, objectives, and requirements. Only consenting participants progressed to the study materials. Participants completed a demographic section (i.e., age, gender identity, and occupation) then advanced to the self-report measures. To reduce order and carryover effects, the Qualtrics randomisation tool rotated section order across participants.

Since the study utilized a cross-sectional design, the researchers also employed methodological devices to minimize common-method variance. Explicitly, to create psychological distance between scales, instructions informed that sections contained separate constructs (Tehseen et al., 2017). This detachment reduced the likelihood of uniform response patterns (Jordan and Troth, 2020). Furthermore, to reduce evaluation apprehension and social desirability bias, study instructions stated that there were no correct answers and encouraged participants to respond with their individual opinions (Podsakoff et al., 2003). Upon completion, a full debriefing statement was presented to all participants.

### Ethics statement

The Health and Education Research Ethics and Governance Committee at Manchester Metropolitan University granted ethical approval (Project ID: 79268).

### Data analysis

Prior to analysis, data were screened for missing data, normality, and multicollinearity. No missing data were present; therefore, no imputation or missing data procedures were required. Item scoring followed published scoring protocols for each instrument. One item from the RPBS was reverse coded prior to computation of total scores. In addition, RPBS item responses were transformed from the original 1–7 response format to a 0–6 metric to facilitate consistency with previous Rasch-scaled applications of the instrument. Total scale scores were subsequently computed, with higher scores reflecting greater levels of the construct assessed. Normality was assessed using skewness and kurtosis statistics, and multicollinearity was evaluated using zero-order correlations.

Analyses were conducted using JASP (Version 0.18.3; JASP Team, 2024). Descriptive statistics and zero-order Pearson correlations were first calculated to examine distributions and bivariate relationships among variables. All variables were treated as continuous indicators.

Network estimation and visualization were conducted using the JASP Network module, which implements procedures based on the *qgraph* and *bootnet* frameworks in R. A Gaussian Graphical Model (GGM) was estimated, wherein edges represent regularized partial correlations between variables after controlling for all remaining nodes in the network (Epskamp et al., 2018).

To estimate a sparse and interpretable network, the graphical least absolute shrinkage and selection operator (graphical LASSO; Friedman et al., 2008) was combined with the Extended Bayesian Information Criterion (EBIC) model selection procedure (Foygel and Drton, 2010). The EBIC hyperparameter was set to the default conservative value of γ = 0.50. This procedure regularizes small partial correlations toward zero, reducing the likelihood of spurious associations and improving network interpretability. Because the network was estimated from partial correlations, variables were effectively analyzed on a standardized metric, allowing comparisons across measures with differing response scales. Accordingly, centrality indices were derived from standardized network parameters rather than raw scale totals.

Centrality indices were computed to assess the relative importance of nodes within the network. These included strength, closeness, betweenness, and expected influence. Strength reflects the summed absolute edge weights connected to a node, closeness reflects the average distance from one node to all others, and betweenness indexes the extent to which a node lies on the shortest paths between other nodes (Opsahl et al., 2010). Expected influence was additionally calculated because it retains the sign of edge weights and is considered particularly informative in psychological networks containing both positive and negative associations (Robinaugh et al., 2016).

Network visualization employed a force-directed Fruchterman–Reingold layout algorithm, whereby nodes with stronger and more numerous associations are positioned closer together. Edge thickness represents the magnitude of the partial correlation, while edge color denotes the direction of the association (positive or negative). This analytical approach allows for the identification of unique relationships among variables and the detection of key nodes within the network, providing insight into the structure of associations among paranormal belief, wellbeing, and emotional constructs.

## Results

### Descriptive statistics

Preliminary screening indicated acceptable univariate normality, with all skewness and kurtosis values falling between −1 and +1. Inspection of zero-order correlations revealed no evidence of problematic multicollinearity, as no correlations exceeded *r* = 0.90. Descriptive statistics and zero-order correlations are presented in Table 1. Participants reported moderate levels of Optimism (*M* = 4.43, SD = 1.52), Pessimism (*M* = 3.63, SD = 1.61), and wellbeing indicators, including Flourishing (*M* = 40.40, SD = 8.79) and Thriving (*M* = 35.29, SD = 7.95). Positive Affect was higher on average (*M* = 21.18, SD = 4.91) than Negative Affect (*M* = 14.64, SD = 4.77). Paranormal Belief (PB) showed sizable variability (*M* = 59.11, SD = 34.50).

**TABLE 1:** Descriptive statistics and correlations among study variables.

Variable	*M*	SD	1	2	3	4	5	6	7
1. Paranormal belief	59.11	34.50	–	–	–	–	–	–	–
2. Optimism	4.43	1.52	0.18**	–	–	–	–	–	–
3. Pessimism	3.63	1.61	0.03	−0.64**	–	–	–	–	–
4. Positive emotion	21.18	4.91	0.04	0.61**	−0.52**	–	–	–	–
5. Negative emotion	14.64	4.77	0.15**	−0.43**	0.46**	−0.68**	–	–	–
6. Flourishing	40.40	8.79	0.05	0.61**	−0.49**	0.76**	−0.60**	–	–
7. Thriving	35.29	7.95	0.07*	0.64**	−0.50**	0.80**	−0.60**	0.89**	–

*P < 0.05, **p < 0.001.

PB was weakly but significantly positively correlated with Optimism (*r* = 0.18, *p* < 0.001), Negative Affect (*r* = 0.15, *p* < 0.001), and Thriving (*r* = 0.07, *p* = 0.006), but was not significantly associated with Pessimism, Positive Affect, or Flourishing. In contrast, wellbeing variables showed strong intercorrelations, particularly between Thriving and Flourishing (*r* = 0.89, *p* < 0.001), and between Positive Affect and Thriving (*r* = 0.80, *p* < 0.001) and Flourishing (*r* = 0.76, *p* < 0.001).

### Network analysis

The estimated network consisted of seven nodes and 18 of 21 edges, indicating a dense structure with low sparsity (0.143) with substantial interconnectivity among the variables. Centrality indices were examined to identify the relative importance of each node within the network (see Table 2). Thriving emerged as the most central node, showing the highest strength and expected influence, specifying that it was strongly and positively connected to other network constructs. Optimism demonstrated the highest betweenness, signifying that it may serve as a bridge connecting different clusters of variables. In contrast, Pessimism and Negative Affect produced negative expected influence values, demonstrating that they were associated with adverse relationships within the network.

**TABLE 2:** Centrality indices for network variables.

Variable	Betweenness	Closeness	Strength	Expected influence
Thriving	1.19	1.17	1.30	1.49
Flourishing	−0.89	−0.86	0.11	0.83
Optimism	1.60	0.57	0.89	−0.45
Pessimism	−0.89	−1.29	−0.54	−1.23
Paranormal belief	−0.47	−0.97	−1.56	0.63
Negative emotion	−0.47	0.76	−0.72	−0.93
Positive emotion	−0.06	0.63	0.52	−0.33

Strength reflects the sum of absolute edge weights connected to each node. Expected influence reflects the sum of edge weights, accounting for the direction (positive or negative) of associations.

PB exhibited low centrality, particularly in terms of strength and closeness, revealing that it occupied a peripheral position within the network. This suggests that PB is not strongly embedded within the broader system of wellbeing and emotional constructs. However, its expected influence connections with other variables, although limited in magnitude, were positive.

Collectively, while wellbeing and affective variables form a highly interconnected core (centered around Thriving and Optimism), PB remains comparatively independent. Its peripheral position, combined with weak but positive associations, denotes a modest and indirect role within the network.

**FIGURE 1:**
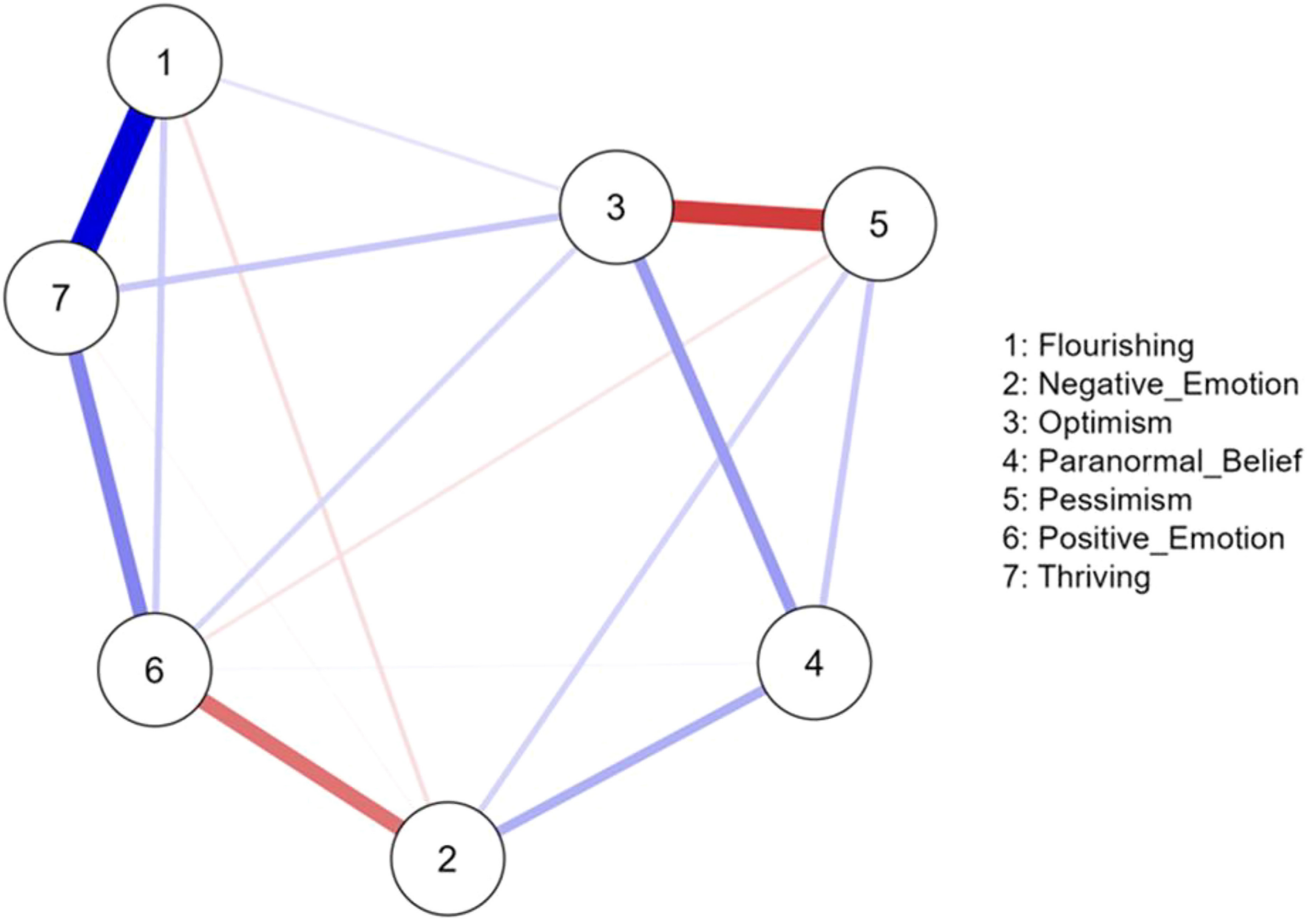
Network structure of relationships among paranormal belief, wellbeing, and emotional variables. Blue edges represent positive associations, and red edges represent negative associations. Thicker edges indicate stronger relationships. Nodes positioned closer together are more strongly connected.

Inspection of the network structure (Figure 1) revealed two primary clusters of variables. A positive wellbeing cluster characterized by strong positive edges among Thriving, Flourishing, and Positive Affect, and a negative cluster with Pessimism showing positive associations with Negative Affect and negative associations with Positive Affect, reflecting an opposing pattern of relationships. Optimism occupied a bridging position, showing positive connections with wellbeing variables and negative associations with Pessimism, thereby linking the positive and negative clusters. This was consistent with its high betweenness centrality.

PB demonstrated weak and selective connections within the network. It was only weakly connected to Optimism, Negative Affect, and, to a lesser extent, Thriving; showing no direct connections with Flourishing or Positive Affect. These findings reveal that PB was not embedded within the core wellbeing structure but instead occupied a peripheral position, with limited direct influence on other variables.

## Discussion

The present study employed network analysis (NA) to provide a representation of relationships between paranormal belief (PB), psychological wellbeing, and adaptive functioning. This approach by mapping the positional importance of PB within a wellbeing network specified which variables act as central or bridge nodes. Within the wellbeing network, PB did not occupy a central role, instead PB operated as a peripheral interpretive framework that indirectly connected with eudaimonic functioning via optimism and affective processes.

Moreover, analysis revealed a highly interconnected network characterized by a eudaimonic hub, defined by strong internal density between Flourishing, Thriving, and Positive Affect. Within this system, Thriving emerged as the central construct, showing the highest strength and expected influence, while Optimism demonstrated the highest betweenness, signifying its role as a key bridge connecting the positive and negative components of the system. Conversely, Pessimism and Negative Affect were associated with a pattern of negative influence, which was distinct from the eudaimonic core.

As expected, PB occupied a peripheral position within the network, demonstrating weak and selective associations with other variables. Specifically, PB demonstrated small positive links with Optimism, Negative Affect, and Thriving and was not directly connected to Flourishing and Positive Affect. These associations imply that while PB is independent of the central wellbeing structure, it is functionally integrated into the network via its association with Optimism. Thus, by enhancing the capacity to maintain positive future-oriented expectations, PB acts as an interpretive lens that bridges the gap between belief and adaptive functioning. Specifically, PB may provide a sense of control that promotes future-oriented expectations, thereby facilitating goal setting and adaptive behavior.

Beyond its structural position, the present findings can be interpreted within broader cognitive and motivational frameworks that conceptualize belief systems as tools for managing uncertainty and existential threat. From this perspective, PB may operate as a form of compensatory control, whereby individuals endorse supernatural explanations to restore a sense of order and predictability when faced with ambiguity or lack of control (Kay et al., 2009; Whitson and Galinsky, 2008). This aligns with the observed association between PB and optimism, suggesting that belief in external or unseen forces may support positive future-oriented expectations even in the absence of direct control over outcomes.

Additionally, the peripheral positioning of PB within the network is consistent with dual-process models of cognition, which propose that intuitive, experiential thinking styles underlie supernatural belief endorsement, while analytic processing constrains it (Evans and Stanovich, 2013; Pennycook et al., 2012). Within this framework, PB does not need to be centrally embedded within wellbeing systems to exert psychological influence; rather, it may function as a flexible interpretive resource that individuals draw upon in specific contexts. This interpretation is further supported by research linking PB with meaning-making processes, where supernatural beliefs provide coherent narratives that enhance perceived purpose and existential coherence (Bering, 2006; Park, 2010). Consequently, PB may contribute to wellbeing not through direct structural integration, but by complementing established psychological mechanisms involved in sense-making and adaptive coping.

These results demonstrate PB’s modest and indirect role within the broader system of psychological health. This is commensurate with the notion that PB facilitates the navigation of existential challenges by aligning with domains of sense-making and meaning. These findings contrast with foundational research, such as the deficit model (Alcock, 1981), which contends that PB is a maladaptive trait. Instead, they concur with contemporary research viewing PB within an adaptive cognitive framework (Dagnall et al., 2025c; Irwin, 2009).

While the current network focused on indicators of flourishing and thriving, the observed integration of PB into these positive domains aligned with previous evidence that PB functions as a stable, non-detrimental cognitive framework (Dagnall et al., 2024; Drinkwater et al., 2024). Particularly, PB is affiliated with sense-making and adaptive psychological functioning (Dagnall et al., 2025b).

## Limitations

Although the observed network structure supported a eudaimonic framework for PB, it is important to note limitations. An issue was the use of cross-sectional design, which by capturing scores at only one point in time, restricted the ability to draw causal conclusions. To address this, future studies should employ longitudinal designs that permit the tracking of psychological changes across extended durations.

A further constraint was potential bias arising from the use of self-report measures. Although study instructions used methodological devices to reduce common method variance, evaluation apprehension, and social desirability these did not eliminate conscious or unconscious reporting biases (i.e. self-insight or the tendency to provide socially acceptable responses). Acknowledging this, subsequent studies should triangulate responses with objective behavioral measures (i.e., laboratory-based tasks or observable performance metrics) and/or informant-based reports (i.e., collecting data from close associates to provide an external perspective on the participant’s traits and behaviors).

The RPBS was used as a global measure. Though this approach was consistent with and enables comparisons with previous studies, the use of total scores obfuscates unique valence affiliated with specific supernatural phenomena. Furthermore, while PB was treated as a single node, future work might find that specific belief types (e.g., traditional religion vs. psi) connect to the eudaimonic hub differently. For instance, eudaimonic associations linked to spiritualism may differ from more threatening or high arousal beliefs (i.e., poltergeists). Thus, while the current study provided a robust high-level network, future research should assess how valence associated with explicit PB dimensions relates to subjective wellbeing.

This study utilized short-form scales, such as the SOP2, to measure subjective wellbeing. Although, these instruments were psychometrically robust and well-suited for large-sample online recruitment, they lack the depth required to capture the nuances of future-oriented expectations. Explicitly, they fail to distinguish between passive optimism and active goal-striving and overlooked the complexities of eudaimonia (i.e., autonomy and environmental mastery) (Ryff, 1989). To achieve this, future investigations should include multidimensional measures (e.g., the Ryff Scales of Psychological Wellbeing). These instruments will provide granular insights into how subcomponents of wellbeing interact with PB. By incorporating comprehensive measures alongside current structural findings, future studies will advance from global understanding to precise mapping of the wellbeing-belief connection.

In addition to these conceptual constraints, the use of abbreviated instruments has implications for structural estimation within network models. This is particularly true when short-form measures like the SOP2 are used in network analytic frameworks. Although such instruments possess established validity and reliability and are widely used in large-scale survey research (Nießen et al., 2022), their brevity introduces potential measurement limitations. Since network models treat observed variables as nodes and do not explicitly model latent measurement error, constructs assessed with fewer items may be susceptible to unreliability, which can attenuate edge weights and bias centrality estimates. Consequently, interpretations of node strength and positioning derived from short-form measures should be made with appropriate caution.

Nevertheless, the use of ultra-short measures is justified in high-burden research contexts which threaten data quality. In studies characterized by repeated measurement, large samples, or cognitively demanding survey batteries, brief instruments reduce burden, improve completion and retention rates, and minimize careless responding (Pank et al., 2025; Schäfer et al., 2023). This approach aligns with broader developments in network analysis, where researchers frequently employ abbreviated scales to include multiple constructs without overextending the model (Epskamp et al., 2018). For example, measures such as the PHQ-2 for depressive symptoms (Kroenke et al., 2003) and the GAD-2 for anxiety (Kroenke et al., 2007) are commonly used as efficient indicators of core symptom domains.

Within this framework, the SOP2 provides a parsimonious representation of dispositional outlook that supports its integration into complex systems models. Indeed, the scale’s use has facilitated the examination of relationships between optimism–pessimism and broader psychological constructs (e.g., future-oriented concerns and technology-related anxiety) (see Khoury et al., 2025). In addition, SOP2 brevity reduces redundancy and mitigates risks associated with multicollinearity or over-clustering of conceptually related items, which can inflate centrality estimates and obscure network structure. This is consistent with methodological guidance in network psychometrics that cautions against overly granular item-level representations (Khoury et al., 2025). Accordingly, while future studies should replicate these findings using multi-item measures to enable sensitivity analyses, the SOP2 remains a suitable and widely used instrument for large-scale network research (e.g., Beard et al., 2016).

Cultural context is another critical consideration. As this study used a UK-based sample rooted in Western scientific rationalism, the adaptive functions of PB may manifest differently in non-Western, secular, or highly religious societies. Acknowledging this, to establish the generalizability of the findings, future research should compare outcomes across a range of cultures. This will determine if the observed eudaimonic-PB network represents a universal psychological mechanism for subjective wellbeing or if it is culturally specific.

To extend the hypothesized model ensuing investigations should include additional PB and wellbeing related factors (e.g., transliminality and schizotypy). Though, not the primary focus within this paper, the absence of these constructs within the current network leaves the psychological catalysts of the belief-wellbeing interface unaccounted for. For instance, transliminality, with its permeable mental boundaries and openness to imagery, may act as a dispositional catalyst for eudaimonic wellbeing (e.g., environmental mastery and personal growth) observed in this network. Similarly, accounting for positive schizotypy would clarify whether the eudaimonic-PB connection is a universal feature of belief or is specifically mediated by propensity for anomalous perception. Integrating these variables will determine if eudaimonia is a direct byproduct of PB or if both emerge from an underlying cognitive architecture predisposed to deriving meaning from the paranormal.

A further constraint was that these results cannot be generalized to clinical populations, where PB co-occurs with heightened psychiatric distress or maladaptive schizotypy. In such contexts, the relationship between PB and wellbeing shifts from a supportive, meaning-making framework to one characterized by cognitive disorganization or functional impairment. Future research is therefore required to determine the diagnostic thresholds at which the eudaimonic benefits of PB are superseded by clinical symptomology.

## Conclusion

In summary, the present study provides a network-based perspective on the role of paranormal belief (PB) within psychological wellbeing. The findings demonstrate that PB occupies a peripheral role characterized by weak yet positive associations with optimism and affective processes. This structural positioning suggests that PB does not directly drive wellbeing; rather, it operates as an interpretive cognitive framework that supports adaptive functioning indirectly, particularly through future-oriented expectations. These results challenge deficit-based accounts of PB and instead support contemporary views that conceptualize it as a context-dependent and non-pathological aspect of cognition. By situating PB within a broader network of hedonic and eudaimonic variables, the study advances understanding of how belief systems contribute to meaning-making and psychological adaptation. Future research should build on these findings by employing longitudinal and cross-cultural designs to further clarify the conditions under which PB enhances or undermines wellbeing.

## Data Availability

The raw data supporting the conclusions of this article will be made available by the authors, without undue reservation.
